# Genetics and genomic medicine in Mali: challenges and future perspectives

**DOI:** 10.1002/mgg3.212

**Published:** 2016-03-17

**Authors:** Guida Landouré, Oumar Samassékou, Mahamadou Traoré, Katherine G. Meilleur, Cheick Oumar Guinto, Barrington G. Burnett, Charlotte J. Sumner, Kenneth H. Fischbeck

**Affiliations:** ^1^ Service de NeurologieCentre Hospitalier Universitaire du Point “G”BamakoMali; ^2^ Neurogenetics BranchNational Institute of Neurological Disorders and Stroke (NINDS)National Institutes of Health (NIH)BethesdaMaryland; ^3^ Manitoba Institute of cell BiologyUniversity of ManibotaWinnipegCanada; ^4^ Service de cytogenetique et de biologie reproductiveInstitut National de Recherche en Santé Publique (INRSP)BamakoMali; ^5^ Tissue Injury BranchNational Institute of Nursing Research (NINR)NIHBethesdaMaryland; ^6^ Departments of Anatomy, Physiology and GeneticsUniformed Services University of the Health Sciences (USUHS)BethesdaMaryland; ^7^ Department of NeurologyJohns Hopkins HospitalBaltimoreMaryland

## Abstract

Genetics and genomic medicine in Mali: challenges and future perspectives.

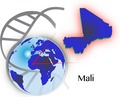

## Introduction

**Figure 1:**
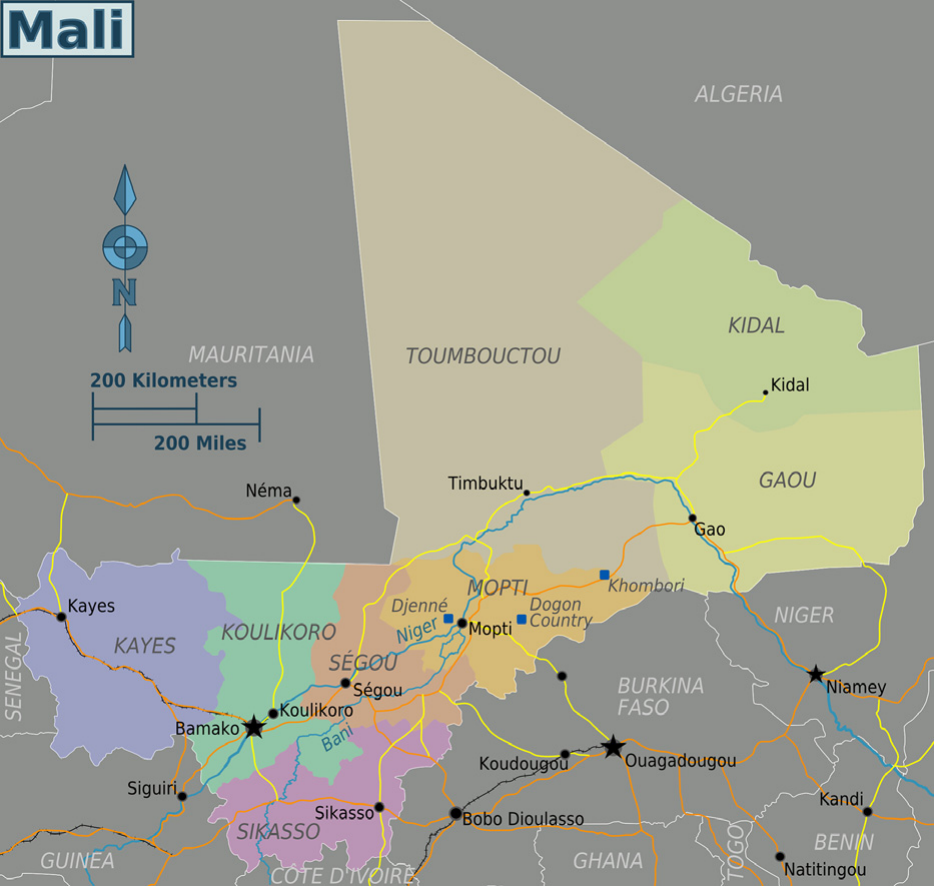
The map of Mali showing surrounding countries and historical sites such as Timbuktu, Djenne, and the Dogon country (mapscity2016.blogspot.com). Each color represents a region where live mostly homogeneous and ethnically similar populations.

Mali is a landlocked country located in the center of West Africa and is surrounded by seven other countries (Fig. 1). With a territory of 1,220,190 sq km and a population of 15,302,000, the country has a young population; 47% are under 15 years of age (WHO, 2013). The country is classified as a low‐income country according to the World Bank's income classification. As such, it relies on international donations or aid programs to operate many of its key sectors, including healthcare. Mali is an ethnically and culturally diverse country, and its subpopulations have a long tradition of intraethnic and consanguineous marriages. This results in homogeneous cluster populations with typical phenotypic characteristics and increased prevalence of recessive disorders in some parts of the country. In fact, there are at least 14 different ethnic groups each speaking a different language. However, about 80% of Malians speak the national language that is Bambara, also spoken in five neighboring countries. Besides these, Malians speak foreign languages including French that is the official language and English which is mostly spoken by some businessmen and skilled people such as researchers.

Genetic and genomic studies in Mali have been limited, leading to an underestimation and neglect of genetic diseases and the genetic contribution in common diseases. This may be due to social factors surrounding these diseases, limited resources preventing patients from seeking care, and a lack of infrastructure and health professionals. In fact, the low literacy rate in Mali, as in many developing countries, may foster a low understanding of basic genetic concepts by the general population. For instance, Malians often consider genetic diseases to be a result of bad fate, which leads to their stigmatization. In addition, limited resources have led authorities to place less emphasis on education in genetics, often mistakenly considered by medical students as a difficult science and not within their reach.

Genetic diseases often cause premature death, severe disability and loss of productivity, resulting not only in high health care costs, but also in high loss of disability‐adjusted life years (DALYs). While these numbers are estimated in more developed countries such as in the United States and Europe (Bonkowsky et al. 2010; Gustavsson et al. 2011), the burden of genetic diseases remains largely unrecognized in developing countries, in part because of the high prevalence and mortality of infectious diseases. Nevertheless, the lack of a highly developed healthcare system and the low per‐household income make this burden even greater in developing countries such as Mali. Health policies in the developing world, and Mali in particular, have focused on preventing and treating communicable diseases and have not prioritized investing in research and medical care on genetic diseases. Recurrent infectious disease outbreaks such as cholera, meningitidis, and the Ebola virus disease make it even harder for noncommunicable disorders to be recognized as a public health concern. Genetic disorders have been widely studied in more developed countries; however, these diseases are not well characterized in sub‐Saharan African populations, and their underlying genetic causes are therefore largely unknown.

Although Africa harbors a majority of infectious diseases, studies on the genetic susceptibility to infectious diseases or resistance to treatment have been scarce on the continent. While searching for resistance variants in the treatment of HIV/AIDS and tuberculosis has become a standard practice in developed countries, in Mali, as in several parts of Africa, genetics and genomic medicine of infectious diseases is still limited to few research laboratories.

## Health care system

### General healthcare

Based on a birth registration coverage of 81%, the number of live births in Mali is 723.3 per 1000, and the mortality rate is high compared to other countries (WHO, 2013). The leading causes of infant mortality are infectious diseases, including lower respiratory infections, diarrheal diseases and malaria. The number of children born per woman is 6.8, however, many do not survive past 5 years. With a gross domestic product (GDP) per capita of approximately $1700, Malians have minimal funds to spend on healthcare. In addition, government health expenditures are only 7.1% of GDP. This translates into a low physician density of 0.08 physicians per 1000 people and low hospital bed density of 0.1 beds per 1000 people.

Since the mid 1980s, Mali has adopted a health care system based on community engagement. This has led to the implementation of reference healthcare and community healthcare centers in each district. With this policy, Mali has improved healthcare delivery to local communities and has surpassed other WHO region countries in health services, leading to an improvement in some indicators. For example, the number of births attended by skilled healthcare provider is above the WHO mean for the region, and life expectancy has increased by 9 years in the past 10 years or so, while it has increased 7 years in other WHO region countries.

In 2010, Mali implemented obligatory, affordable health care insurance for government and registered private employees, which represent just 20–22% of the population. Unfortunately, many of the eligible individuals have not enrolled. People with little or no income also benefit from health care coverage through government subsidies. However, the process is cumbersome and does not cover all medical expenses. Nevertheless, these programs are promising steps for a country where health insurance had been limited to employees of some private companies only.

Despite the progress made in recent years regarding healthcare services, Mali is still lagging behind in terms of genetic healthcare delivery. Until recent years, there were few medical residencies in certain specialties in the country. Most medical doctors went to neighboring countries to get further training at their own expense. With an overall education expenditure of 4.8% of the GDP (https://www.cia.gov/library/publications/the-world-factbook/geos/ml.html), the majority of funds go to subsidize primary education. Until now, only a subset of medical doctors is sponsored by the government to get their specialty degree. Almost all specialists are concentrated in the capital city Bamako or in regional centers, and the recent unrest in the northern 2/3 of the country has left the populations of this region with almost no highly skilled health care practitioners. Despite the recent expansion of residency programs in almost all medical specialties, there still is no such program in genetics. This is due to the lack of qualified trainers, but also to health policies focusing primarily on common and communicable diseases.

### Medical genetics

Genetic testing has become a standard procedure in developed countries, and it has helped to substantiate clinical diagnoses and management as well as identify disease carriers, thus offering the opportunity to reduce the incidence of recessive disorders. However, to date there is no medical genetics clinic in Mali. The practice of medical genetics has been largely limited to sickle cell disease (SCD). Although it is one of the most common inherited diseases in the world, SCD was recognized by WHO as public health concern only in 2006. Mali has a prevalence of 3% with 5–6000 newborns with SCD each year (Sangho et al. 2009). Still, the disease remains neglected and is not recognized as genetic by the majority of population (Sangho et al. 2009; Diallo and Guindo 2014). Only a portion of the population has access to the diagnosis of SCD, and education about the inheritance of the disease is not provided because of the lack of specialists in the field. Moreover, prenatal and early postnatal diagnostic of SCD cannot be done in Mali because of the lack of a clinical genetic laboratory. Mali also does not have any genetic counselors or genetic counseling programs. This role is fulfilled by hematologists and other physicians who see patients with genetic disorders. Diagnosis by hemoglobin electrophoresis is widely offered in the capital city but in only a few of the regional health centers. The molecular diagnosis of other genetic diseases often relies on partnerships with external investigators. Those people who are wealthier may obtain genetic testing overseas.

**Figure 2:**
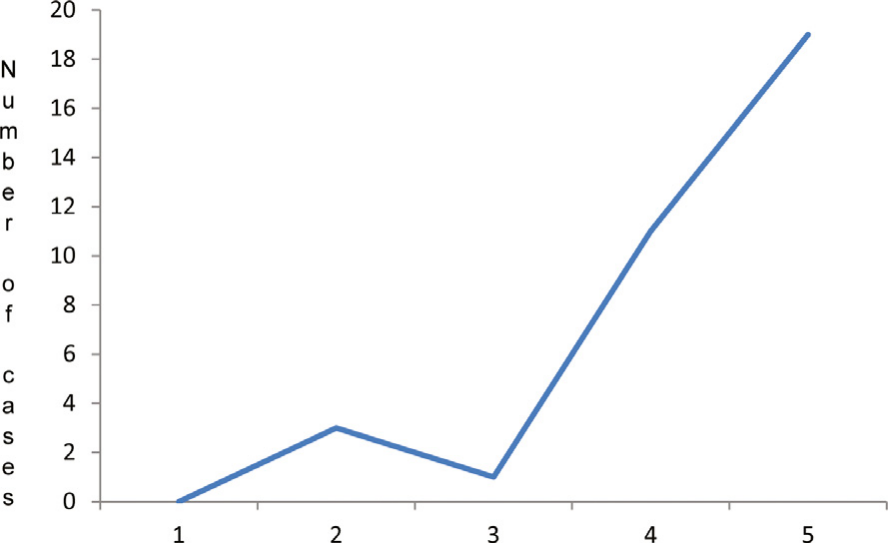
The progression of the number of molecular diagnoses done in the country in the last decades. Note the sharp increase after the establishment of the collaboration between NINDS and Malian investigators.

Before 2007, the few case reports in the literature of molecular diagnoses of single gene diseases in Mali mostly involved chromosomal abnormalities. After training in Europe, M. Traoré started cytogenetic studies in his new laboratory and reported a few cases of chromosomal abnormalities including trisomy 8, 13 and 21 (Touré and Traoré 1997; Traoré et al. 1997a,b). Since then, no other such studies in local laboratories have followed because of lack of funding and government support. The only exceptions are those done by Malian researchers with temporary collaborations with external investigators or by European researchers on Malian families in the diaspora (Traoré et al. 2004; Maystadt et al. 2007). In 2002, after a Malian neurologist invited investigators from the National Institute of Neurological Disorders and Stroke (NINDS) of the National Institutes of Health (NIH) for a field visit, a collaborative, larger scale genetic study of hereditary neurological disorders was undertaken. Since then, the known number of molecular diagnoses done in the country has sharply gone up (Fig. 2).

Other departments, such as the Hematology and Oncology Department in Bamako, have also reported some genetic studies on breast cancer as a part of research studies. However, no regular medical genetics services are offered in this department (Ly et al. 2012, 2013). Although certain cancers require genetic classification for efficient treatment, this practice has not yet been introduced in Mali. Populations with African ancestry have been shown to have a higher prevalence of some types of cancers. Therefore, there is a need to boost cancer research in these regions to improve understanding of these diseases and develop new therapeutic approaches that may be applied to other populations.

### Genetic education

As mentioned previously, to date there is no genetics degree program in Mali. Genetic teaching is limited to the few hours taught in high school and some university programs, and covers only basic genetics concepts. Therefore, new genetic technologies and concepts are often unknown to medical practitioners. Until recently, Mali had only one genetic specialist, who trained in Europe. However, this number has increased more recently and we have now four trained geneticists on site and one other, who shares his time between Mali and Canada. The existing collaboration between NINDS and Malian researchers has allowed two Malians to complete a PhD degree in molecular biology through the NIH exchange visitor program. This new dynamic has boosted genetic research in Mali with several genetic research protocols in place or under development. This also allows for a unique opportunity to train students in genetics and molecular biology. The recent development of genetic research in Africa will generate a large amount of data from African populations; pointing to the need and the opportunity to train more scientists in genetics and bioinformatics and to reduce the large existing gap between Africa and other continents in regard to genomic medicine. It is important for Mali to implement a genetic training program soon to meet the global challenge and demand for genomic and genetic medicine.

## Genetic research

### Existing genetic research

Previous studies have found that the vast majority of genetic research has been done in populations of European descent; only a fraction of genetic research has been done in African populations (Rosenberg et al. 2010). Mali is no exception. Although the first report on genetic diseases in Mali dates back to 1970, with a description of sickle cell disease (Dufrenot and Legait 1970), it is only since the mid 1990s that genetic research truly started in the country. However, most of that research focused on malaria, and mostly on mosquito genetics. In fact, in the late 1980s, the National Institute of Allergy and Infectious Diseases (NIAID) of the NIH built a malaria research facility at the Medical School of Bamako, which to this day performs world‐class research and makes breakthroughs in the treatment and management of malaria. Among many studies, for example, Malian researchers in collaboration with researchers at NIH have shown that mutations in the Plasmodium gene confer resistance to some antimalarial drugs, whereas other mutations increase the susceptibility to other antimalaria drugs (Plowe et al. 1997; Djimdé et al. 2001; Ouattara et al. 2015). This work has led to improvements in the treatment of this devastating disease. The NIAID facility has fostered research in other topics relevant to local public health including tuberculosis, HIV, and filariasis.

In 2007, as part of the ongoing collaboration between Malian researchers and the Neurogenetics Branch of the NINDS, the Neurology Department of the Teaching Hospital of Point “G” (Bamako, Mali) started screening patients with hereditary neurological disorders. Genetic testing and counseling were performed under a joint protocol approved by the IRBs of both institutions. As the Malian population had never been exposed to genetic testing, the knowledge, attitudes, and beliefs of Malian families with hereditary neurological disorders regarding genetic testing were first assessed in pre‐and posttest questionnaires. The results showed that in general, the majority favored genetic testing and some gained knowledge from genetic counseling (Meilleur et al. 2011). The investigation also led to the clinical characterization of many families with hereditary neurological disorders, some of which were given a molecular diagnosis (Meilleur et al. 2011). Of note, some of these families had characteristic features due to novel mutations in known genes (Traoré et al. 2009, 2011; Meilleur et al. 2011; Landouré et al. 2013a,b), one family had a mutation in a new gene, *C19ORF12* (Landouré et al. 2013a,b), and other families were screened negative for all possible candidate genes; confirming the genetic heterogeneity of the Malian population. Moreover, the variant in *C19ORF12* was found in 3 of 3836 African‐American alleles and not in 8222 European‐American alleles (NHLBI Exome Sequencing Project database: http://evs.gs.washington.edu/EVS/). Interestingly, the same mutation in *C19ORF12* was found in a Brazilian family with some clinical overlap, and haplotype reconstruction suggested that the Malian and Brazilian families may be related (Landouré et al. 2013a,b). This study was interesting in that it not only showed the genetic heterogeneity and specificity of a population of African ancestry, but it also revealed the opportunity to uncover mutations using populations with larger sibships. The same gene was shown to cause another neurodegenerative disease in populations with European ancestry; the clinical difference may be due to genetic or environmental modifiers. Despite these findings, not all families needing genetic testing could be included, and the project was slowed down because of recurring political standoffs. Although far from establishing a survey of these diseases in Mali, this collaboration has given a glimpse of their spectrum in this part of Africa. This study also showed that spinocerebellar ataxia (SCA), myopathies and spastic paraplegia (SPG) may be the most common hereditary neurological disorders, and, except for SCA, recessive diseases are predominant; which is likely due to the high rate of consanguinity. To date, many other families have been clinically characterized and molecular diagnosis established in some families. Among SCAs, SCA2 is the most common and concentrates in a specific region of the country (unpublished), while limb‐girdle muscular dystrophy (LGMD) are the most common myopathies (unpublished) (Table 1).

**Table 1:** Summary of clinically diagnosed Mendelian disorders with some molecular diagnoses in the Malian population in recent years

Type	Subtype	No. of families	Transmission	Gene	Mutation
Muscular disease	LGMD, type 2D	1	AR	*SGCA*	Novel c.574C>T (Arg192X) (Meilleur et al. 2011)
	LGMD, type 2B	1	CH	*DYSF*	2 novel mutations: c.2643 + 1G>A, c.4018G>C (splice site, Gly1340Arg)
	Duchenne muscular dystrophy	2	X‐linked	*DMD*	Novel c.3784delG (1262E>FrameshiftKX20)
	Duchenne muscular dystrophy	2	X‐linked	*DMD*	Novel c.1423G>T (Glu475X)
	Myotonia congenita	1	AD	*CLCN1*	Novel c.1672C>T (Pro558Ser)
	Kearns–Sayre syndrome	1	mtDNA	*mtDNA*	5585 bp deletion
	LGMD	9	AR	N/A	N/A
	LGMD	5	S	N/A	N/A
Epilepsy	Lafora disease	2	AR	*EPM2B*	Novel c.560A>C (His187Pro) (Traoré et al. 2009)
	PME	2	AR	PME panel	Negative
	PME	2	AR	N/A	N/A
Ataxia	Spinocerebellar ataxia, type 2	7	AD	*ATXN2*	Heterozygous CAG repeat expansion
	Spinocerebellar ataxia, type 3	4	AD	*ATXN3*	Heterozygous CAG repeat expansion
	Spinocerebellar ataxia, type 7	4	AD	*ATXN7*	Heterozygous CAG repeat expansion
	Ataxia telangiectasia	1	AR	*ATM*	Novel c.7985T>A (Val2662Asp) (Landouré et al. 2013a,b)
	Spinocerebellar ataxia	1	AD	N/A	N/A
	Spinocerebellar ataxia	7	S	N/A	N/A
	Spinocerebellar ataxia	1	AR	N/A	N/A
CMT	CMTX1	1	X‐dominant	*GJB1*	Heterozygous c.704T>G (Phe235Cys)
	Axonal CMT	4	AR	N/A	N/A
	Axonal CMT	2	AD	N/A	N/A
	CMT	1	S	N/A	N/A
SPG	SPG43	1	AR	*C19orf12*	Novel c.187G>C (Ala63Pro) (Landouré et al. 2013a,b)
	Juvenile SPG	2	AR	*ERLIN2, ALS2*	Negative
	Recessive SPG	5	AR	N/A	N/A
	Dominant SPG	2	AD	N/A	N/A
SMA	SMA type 2	2	AR	*SMN1*	SMN1 homozygous deletion
	SMA	2	AR	N/A	N/A
Huntingon disease	Huntington disease	1	AD	*Htt*	Heterozygous CAG repeat expansion
	Huntington disease phenotype	5	AD	N/A	N/A
Bone disease	Skeletal dysplasia	2	AD	N/A	N/A
	Skeletal dysplasia	1	AR	N/A	N/A
	Bone malformation + mental retardation	1	AD	N/A	N/A

LGMD, limb‐girdle muscular dystrophy; AR, autosomal recessive; CH, compound heterozygous; AD, autosomal dominant; N/A, not available; S, sporadic; SMA, spinal muscular atrophy.

In parallel, Malian researchers, in collaboration with external investigators, have conducted population genetics studies that included populations of different parts of the continent (Seielstad et al. 1999). Malian ethnic groups such as the Dogon were found to have unique genetic features, which differentiate them from other African populations (Tishkoff et al. 2009). In addition, recent studies in the Malian population have suggested that genetic polymorphisms play a role in increased risk for hypertension in a subpopulation, and low spinal muscular atrophy (SMA) carrier frequency in the general population. Taylor et al. (2013) showed the association of a SNP located in the sodium channel *SLC4A5* with high systolic blood pressure in Dogon women (Taylor et al. 2013). This study may serve as a basis for genetic association with high blood pressure (HBP), possibly in African Americans, many of whom originate from this part of the continent. The Dogon are known to have an active lifestyle and a diet low in fat and sugar. Finding this association with HBP may prove significant by helping potentiate the genetic health delivery for populations of the same genetic background. Sangaré et al. (2014) investigated the carrier frequency of the causative genes from SMA, which has been considered one of the most common genetic diseases of infancy and early childhood worldwide. When Sangare et al. studied *SMN1* and *SMN2* copy numbers in more than 600 Malian students from different ethnic backgrounds, they found a very low SMA carrier frequency in Mali of 1/209 (Sangaré et al. 2014) compared to populations with European or Asian descent where the carrier frequency is 1/30–1/50 (Ogino et al. 2004). Sangaré et al. (2014)also found Malians are more likely to have ≥3 *SMN1* copies than Eurasians. A similar result was found when other African populations from Nigeria and Kenya were assessed. The same study found that Malians have lower copy numbers of the SMA disease modifying gene, *SMN2*. Although higher *SMN1* copy number did not translate into increased SMN mRNA, this opens a path to further our understanding of the apparently low SMA frequency in Mali. In addition, no correlation was found with malaria severity, thus arguing against a possible protective effect of high *SMN1* copy levels. It is still possible that other endemic diseases or environmental cofactors may be causing these high *SMN1* levels. Further functional studies comparing African and Eurasian samples may improve our understanding of the disease mechanism and lead to new therapeutic approaches.

### Research opportunities

Recent advances in genetic health care delivery in the developed world have made disparities between developing and developed countries greater, but these disparities may be mitigated by addressing genetic health care needs and the specific deficits of developing countries. Genetic diseases are often debilitating and untreatable disorders that result in a heavy psychosocial burden, especially in Africa. Although numerous genes have now been identified with mutations for these diseases, the genetic basis of many clinically characterized genetic disorders is not yet established. New genotyping and sequencing technologies such as whole exome sequencing have increased the speed of DNA sequencing, and the cost of these technologies has dropped significantly. This has resulted in rapid identification of the causes of hereditary diseases at a relatively low cost. While the sequencing of the first human genome took approximately 15 years and cost about 1 billion US dollars, the cost and time of performing this sequencing have decreased to less than 1500 US dollars and 1 day. With the cost expected to decrease further, there is hope that African researchers will have the ability to conduct large genomic studies.

**Figure 3:**
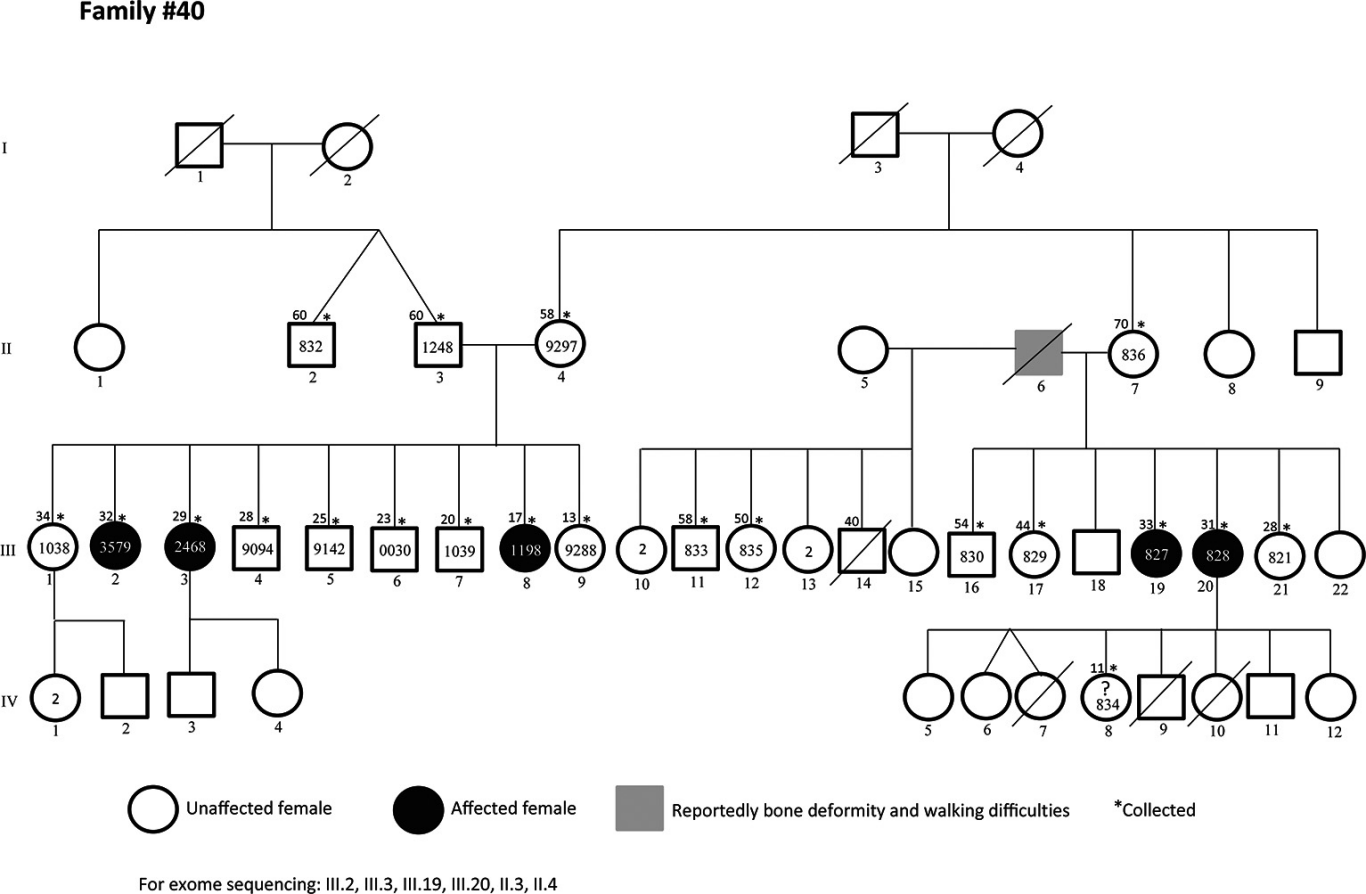
Family with LGMD phenotype negative for all known muscle genes testing. The large number of siblings is an opportunity to find quickly the underlying genetic defect. Asterisks represent subjects with available samples.

**Figure 4:**
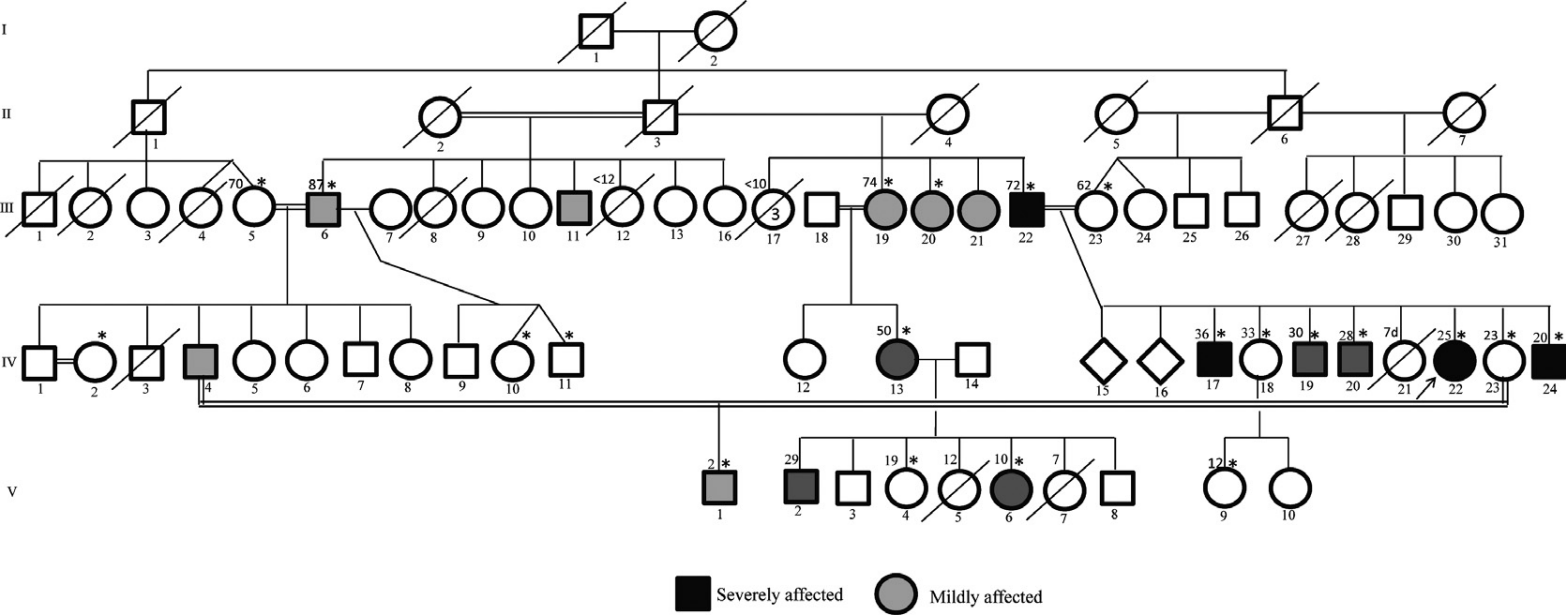
A large family with bone deformities associated with mental retardation and psychiatric symptoms of varied penetrance. Asterisks represent individuals with available samples.

Malians have a high rate of intraethnic and consanguineous marriage, resulting in increased prevalence of autosomal recessive diseases. In a study on a sample of about 600 Malian students from different ethnic backgrounds, 27% reported consanguinity including 17% of parental first cousin marriage (Sangaré et al. 2014). However, these numbers could be higher in some ethnic groups. While family based genetic studies are often limited in developed countries due to small sibships, the average fertility rate in Mali, which is over 6 births per woman, offers a unique opportunity to find new disease genes or mutations that can then be studied in other populations (Figs. 3, 4).

Although the African Union has suggested African countries invest 1% of their GDP into research during their 2006 summit, this has not been fully translated into action yet (http://www.sarpn.org/documents/d0001850/AU_2006_Sudan_Council_Decisions_Khartoum.pdf). While the overall pace of government intervention in research has been slow in Africa, leaders such as Mauritian President Ameenah Gurib‐Fakim are increasingly advocating for more investment in research (http://www.theguardian.com/global-development-professionals-network/2015/sep/30/african-governments-must-invest-in-science-and-research). Current advances in genomic medicine propose the delivery of individualized treatment according to an individual's genetic background. This initiative, entitled Precision Medicine and supported by the US government, will lead to the sequencing of millions of human genomes and will substantiate treatment of several diseases. To date, no known Malian genome is published. If no effort is made by African governments, the disparities in genetic health will be similar to those seen with other new technologies. Few research projects are funded in Mali, and except for SCD, no genetic disease has been funded by the Malian government.

Nevertheless, Malian researchers are increasingly obtaining funding from international funding bodies, including NIH, Welcome Trust (UK), and nongovernmental organizations such as The Bill and Melinda Gates Foundation (Wonkam and Mayosi 2014). More recently, a major program funding African researchers called “Human Heredity and Health in Africa” (H3Africa) was initiated by NIH and the Welcome Trust. The aim of this initiative is to support research in Africa by infrastructure development, training of African researchers, and funding research projects on African soil (Rotimi et al. 2014). Malian researchers have received these funds to investigate the genetic defects of hereditary neurological disorders. In addition, other Malian centers have been selected in the H3Africa bioinformatics support network. This new funding will reinforce the ongoing collaboration between Malian and NIH investigators, and allow investigation of other genetic disorders such as epilepsy that constitutes a major a public health issue in this region of Africa (Landouré et al. 2014).

Despite the contribution of these funding opportunities, it behooves African governments to invest more in genetic education and research in order to ensure sustainability and relevance to local needs. African governments could also take advantage of the growing number of geneticists to implement a timely national survey of genetic diseases and develop genetic healthcare delivery.

## Conclusion

With only about 20% of human genes found to have disease‐associated mutations to date (http://www.ncbi.nlm.nih.gov/Omim/) and thousands of phenotypes showing Mendelian inheritance, many more disease genes remain to be identified, especially in unexplored populations. Although several thousand genes have now been associated with diseases, it remains important to find new genes to further our understanding of their function and interactions and to increase our knowledge of common disease mechanisms. Because of the population diversity in Africa and the limited studies done on the continent, genetic and genomic research in Africa will certainly answer many health questions that could not be solved by studying other populations.

Although medical genetics and research studies of genetic diseases are in their infancy in Mali, tremendous progress has been made during this decade to improve genetic research. A strong partnership between Malian scientists and their international collaborators has been the reason for this improvement. To ensure sustainability, the Malian government should be fully engaged to empower genetic training and research funding. With this support, Malian scientists and clinicians would be ready to meet emerging medical genetics and genomic challenges.

## Conflict of Interest

None declared.

## References

[mgg3212-bib-0001] Bonkowsky, J. L. , C. Nelson , J. L. Kingston , F. M. Filloux , M. B. Mundorff , and R. Srivastava . 2010 The burden of inherited leukodystrophies in children. Neurology 75:718–725. 20660364 10.1212/WNL.0b013e3181eee46b PMC2931652

[mgg3212-bib-0002] Diallo, D. A. , and A. Guindo . 2014 Sickle cell disease in sub‐Saharan Africa: stakes and strategies for control of the disease. Curr. Opinion. Hematol. 21:210–214. 10.1097/MOH.0000000000000038 24613937

[mgg3212-bib-0003] Djimdé, A. , O. K. Doumbo , J. F. Cortese , K. Kayentao , S. Doumbo , Y. Diourté , et al. 2001 A molecular marker for chloroquine‐resistant falciparum malaria. N. Engl. J. M. 344:257–263. 11172152 10.1056/NEJM200101253440403

[mgg3212-bib-0004] Dufrenot, , and J. P. Legait . 1970 Distribution of hemoglobin S and C genes in Upper Volta, Mali and Niger. Bull. Soc. Path. Exot. Filiales. 63:606–614. 5537754

[mgg3212-bib-0005] Gustavsson, A. , M. Svensson , F. Jacobi , C. Allgulander , J. Alonso , E. Beghi , et al. 2011 Cost of disorders of the brain in Europe 2010. Eur. Neuropsychopharmacol. 21:718–779. 21924589 10.1016/j.euroneuro.2011.08.008

[mgg3212-bib-0006] Landouré, G. , F. Mochel , K. Meilleur , M. Ly , M. Sangaré , N. Bocoum , et al. 2013a Novel mutation in the ATM gene in a Malian family with ataxia telangiectasia. J. Neurol. 260:324–326. 23142947 10.1007/s00415-012-6738-5 PMC3566581

[mgg3212-bib-0007] Landouré, G. , P. P. Zhu , C. M. Lourenco , J. O. Johnson , C. Toro , K. V. Bricceno , et al. 2013b Hereditary spastic paraplegia type 43 (SPG43) is caused by mutation in C19orf12. Hum. Mutat. 34:1357–1360. 23857908 10.1002/humu.22378 PMC3819934

[mgg3212-bib-0008] Landouré, G. , Y. Maiga , O. Samassékou , K. Nimaga , M. Traoré , and K. H. Fischbeck . 2014 Epilepsy genetics in Africa: challenges and future perspectives. North Afr. Middle East Epilepsy J. 3:5–7. 26413584 PMC4580280

[mgg3212-bib-0009] Ly, M. , M. Antoine , A. K. Dembélé , P. Levy , A. Rodenas , B. A. Touré , et al. 2012 High incidence of triple‐negative tumors in sub‐saharan Africa: a prospective study of breast cancer characteristics and risk factors in Malian women seen in a Bamako university hospital. Oncology 83:257–263. 22964749 10.1159/000341541

[mgg3212-bib-0010] Ly, M. , A. Valent , G. Diallo , F. Penault‐Lorca , K. Dumke , V. Marty , et al. 2013 Gene copy number variations in breast cancer of Sub‐Saharan African women. Breast 22:295–300. 22999459 10.1016/j.breast.2012.07.010

[mgg3212-bib-0011] Maystadt, I. , R. Rezsohazy , M. Barkats , S. Duque , P. Vannuffel , S. Remacle , et al. 2007 The nuclear factor kappaB‐activator gene PLEKHG5 is mutated in a form of autosomal recessive lower motor neuron disease with childhood onset. Am. J. Hum. Genet. 81:67–76. 17564964 10.1086/518900 PMC1950913

[mgg3212-bib-0012] Meilleur, K. G. , S. Coulibaly , M. Traoré , G. Landouré , A. La Pean , M. Sangaré , et al. 2011 Genetic testing and counseling for hereditary neurological diseases in Mali. J. Community. Genet. 2:33–42. 22109722 10.1007/s12687-011-0038-0 PMC3186021

[mgg3212-bib-0013] Ogino, S. , R. B. Wilson , and B. Gold . 2004 New insights on the evolution of the SMN1 and SMN2 region: simulation and meta‐analysis for allele and haplotype frequency calculations. Eur. J. Hum. Genet. 12:1015–1023. 15470363 10.1038/sj.ejhg.5201288

[mgg3212-bib-0014] Ouattara, A. , A. Koné , M. Adams , B. Fofana , A. W. Maiga , S. Hampton , et al. 2015 Polymorphisms in the K13‐propeller gene in artemisinin‐susceptible Plasmodium falciparum parasites from Bougoula‐Hameau and Bandiagara. Mali. Am. J. Trop. Med. Hyg. 92:1202–1206. 25918205 10.4269/ajtmh.14-0605 PMC4458826

[mgg3212-bib-0015] Plowe, C. V. , J. F. Cortese , A. Djimdé , O. C. Nwanyanwu , W. M. Watkins , P. A. Winstanley , et al. 1997 Mutations in Plasmodium falciparum dihydrofolate reductase and dihydropteroate synthase and epidemiologic patterns of pyrimethamine‐sulfadoxine use and resistance. J. Infect. Dis. 176:1590–1596. 9395372 10.1086/514159

[mgg3212-bib-0016] Rosenberg, N. A. , L. Huang , E. M. Jewett , Z. A. Szpiech , I. Jankovic , and M. Boehnke . 2010 Genome‐wide association studies in diverse populations. Nat. Rev. Genet. 11:356–366. 20395969 10.1038/nrg2760 PMC3079573

[mgg3212-bib-0017] Rotimi, C. , A. Abayomi , A. Abimiku , V. M. Adabayeri , C. Adebamowo , E. Adebiyi , et al. 2014 Research capacity. Enabling the genomic revolution in Africa. Science 344:1346–1348. 24948725 10.1126/science.1251546 PMC4138491

[mgg3212-bib-0018] Sangaré, M. , B. Hendrickson , H. A. Sango , K. Chen , J. Nofziger , A. Amara , et al. 2014 Genetics of low spinal muscular atrophy carrier frequency in sub‐Saharan Africa. Ann. Neurol. 75:525–532. 24515897 10.1002/ana.24114 PMC4112719

[mgg3212-bib-0019] Sangho, H. , H. D. Keita , A. S. Keita , F. Y. Diarra , B. Belemou , A. Dia , et al. 2009 Management of sickle cells disease by households in Bamako. Mali Med. 24:53–56. 20093217

[mgg3212-bib-0020] Seielstad, M. , E. Bekele , M. Ibrahim , A. Touré , and M. Traoré . 1999 A view of modern human origins from Y chromosome microsatellite variation. Genome Res. 9:558–567. 10400923 PMC310766

[mgg3212-bib-0021] Taylor, J. Y. , D. Sampson , A. D. Taylor , D. Caldwell , and Y. V. Sun . 2013 Genetic and BMI risks for predicting blood pressure in three generations of West African Dogon women. Biol. Res. Nurs. 15:105–111. 21859746 10.1177/1099800411419026 PMC3288462

[mgg3212-bib-0022] Tishkoff, S. A. , F. A. Reed , F. R. Friedlaender , C. Ehret , A. Ranciaro , A. Froment , et al. 2009 The genetic structure and history of Africans and African Americans. Science 324:1035–1044. 19407144 10.1126/science.1172257 PMC2947357

[mgg3212-bib-0023] Touré, A. , and M. Traoré . 1997 46, XY/45, X/47, XXY chez un homme azoosperme. Mali Med. 11:1–2.

[mgg3212-bib-0024] Traoré, M. , A. Touré , B. Maiga , and A. Ag Mohamed . 1997a Trisomie 13 et 8 en mosaïque chez un enfant polymalformé. Med. Afr. Noire 44:486.

[mgg3212-bib-0025] Traoré, M. , A. Touré , M. S. Traoré , and M. M. Keita . 1997b Etude cytogénétique chez 13 enfants présentant une polymalformation. Mali Med. 11:48–49.

[mgg3212-bib-0026] Traoré, M. , M. Sylla , J. Traoré , T. Sidibé , and C. O. Guinto . 2004 Type 2 Gaucher's disease in a Malian family. Afr. J. Health Sci. 11:67–69. 17298119

[mgg3212-bib-0027] Traoré, M. , G. Landouré , W. Motley , M. Sangaré , K. Meilleur , S. Coulibaly , et al. 2009 Novel mutation in the NHLRC1 gene in a Malian family with a severe phenotype of Lafora disease. Neurogenetics 10:319–323. 19322595 10.1007/s10048-009-0190-4 PMC2758214

[mgg3212-bib-0028] Traoré, M. , T. Coulibaly , K. G. Meilleur , A. La Pean , M. Sangaré , G. Landouré , et al. 2011 Clinical and genetic analysis of spinocerebellar ataxia in Mali. Eur. J. Neurol. 18:1269–1271. 21418439 10.1111/j.1468-1331.2011.03376.x PMC3136651

[mgg3212-bib-0029] WHO , 2013 World Health Organization: Mali Statistics.

[mgg3212-bib-0030] Wonkam, A. , and B. M. Mayosi . 2014 Genomic medicine in Africa: promise, problems and prospects. Genome Med. 6:11. 25031612 10.1186/gm528 PMC3979013

